# A pragmatic, open-label, multi-center, randomized controlled clinical trial on the rotational use of interfaces *vs* standard of care in patients treated with noninvasive positive pressure ventilation for acute hypercapnic respiratory failure: the ROTAtional-USE of interface STUDY (ROTA-USE STUDY)

**DOI:** 10.1186/s13063-023-07560-1

**Published:** 2023-08-13

**Authors:** Rosanna Vaschetto, Cesare Gregoretti, Lorenza Scotti, Nello De Vita, Annalisa Carlucci, Andrea Cortegiani, Claudia Crimi, Alessio Mattei, Raffaele Scala, Eduardo Rocca, Federico Longhini, Gianmaria Cammarota, Giovanni Misseri, Alberto Dal Molin, Sabino Scolletta, Stefano Nava, Salvatore Maurizio Maggiore, Paolo Navalesi

**Affiliations:** 1 Dipartimento Di Medicina Traslazionale, Università del Piemonte Orientale, Via Solaroli, 17, 28100 Novara, Italy; 2 Anesthesia and Intensive Care, Azienda Ospedaliero-Universitaria Maggiore Della Carità, Novara, Italy; 3 Department of Surgical, Oncological and Oral Science (Di.Chir.On.S.), University of Palermo, Palermo, Italy; 4 G. Giglio Foundation, Cefalù, Italy; 5 Dipartimento Di Medicina E Chirurgia, Università Insubria Varese-Como, Varese, Italy; 6 Department of Anesthesia Analgesia Intensive Care and Emergency, University Hospital Policlinico Paolo Giaccone, Palermo, Italy; 7 Respiratory Medicine Unit, Policlinico “G. Rodolico-San Marco” University Hospital, Catania, Italy; 8 Department of Clinical and Experimental Medicine, University of Catania, Catania, Italy; 9 Respiratory Medicine Unit, Azienda Ospedaliera S. Croce E Carle, Cuneo, Italy; 10 Pulmonology and Respiratory Intensive Care Unit, S. Donato Hospital, Arezzo, Italy; 11 Anesthesia and Intensive Care Unit, Department of Medical and Surgical Sciences, “Magna Graecia” University, Catanzaro, Italy; 12 Dipartimento Di Medicina Translazionale, Università Del Piemonte Orientale, Novara, Italy; 13 Dipartimento Scienze Mediche, Chirurgiche E Neuroscienze, Università Degli Studi Di Siena, Siena, Italy; 14 Respiratory and Critical Care Unit, IRCCS Azienda Ospedaliero-Universitaria Di Bologna, Bologna, Italy; 15 Dipartimento Di Medicina Specialistica, Diagnostica E Sperimentale, Università Di Bologna, Bologna, Italy; 16 University Department of Innovative Technologies in Medicine and Dentistry, Università “G. D’Annunzio” Di Chieti-Pescara, Chieti, Italy; 17 Clinical Department of Anesthesiology and Critical Care Medicine, SS. Annunziata Hospital, Chieti, Italy; 18 Dipartimento Di Medicina - DIMED, Università Di Padova, UOC Istituto Di Anestesia E Rianimazione, Azienda Ospedale-Università Di Padova, Padua, Italy

**Keywords:** Noninvasive ventilation, Hypercapnic acute respiratory failure, Pressure ulcers, Rotational use of interfaces

## Abstract

**Background:**

In the last decades, noninvasive ventilation (NIV) has been increasingly used to support patients with hypercapnic and hypoxemic acute respiratory failure. Pressure ulcers are a frequently observed NIV-related adverse effect, directly related to interface type and exposure time. Switching to a different interface has been proposed as a solution to improve patient comfort. However, large studies investigating the benefit of this strategy are not available. Thus, the aim of the ROTAtional-USE of interface STUDY (ROTA-USE STUDY) is to investigate whether a protocolized rotational use of interfaces during NIV is effective in reducing the incidence of pressure ulcers.

**Methods:**

The ROTA-USE STUDY is a pragmatic, parallel arm, open-label, multicenter, spontaneous, non-profit, randomized controlled trial requiring non-significant risk medical devices, with the aim to determine whether a rotational strategy of NIV interfaces is associated with a lower incidence of pressure ulcers compared to the standard of care. In the intervention group, NIV mask will be randomly chosen and rotated every 6 h. In the control group, mask will be chosen according to the standard of care of the participating centers and changed in case of discomfort or in the presence of new pressure sores. In both groups, the skin underneath the mask will be inspected every 12 h for any possible damage by blinded assessors. The primary outcome is the proportion of patients developing new pressure sores at 36 h from randomization. The secondary outcomes are (i) onset of pressure sores measured at different time points, i.e., 12, 24, 36, 48, 60, 72, 84, and 96 h; (ii) number and stage of pressure sores and comfort measured at 12, 24, 36, 48, 60, 72, 84, and 96 h; and (iii) the economic impact of the protocolized rotational use of interfaces. A sample size of 239 subjects per group (intervention and control) is estimated to detect a 10% absolute difference in the proportion of patients developing pressure sores at 36 h.

**Discussion:**

The development of pressure ulcers is a common side effect of NIV that negatively affects the patients’ comfort and tolerance, often leading to NIV failure and adverse outcomes. The ROTA-USE STUDY will determine whether a protocolized rotational approach can reduce the incidence, number, and severity of pressure ulcers in NIV-treated patients.

**Trial registration:**

ClinicalTrials.gov NCT05513508. Registered on August 24, 2022.

## Administrative information

Note: the numbers in curly brackets in this protocol refer to SPIRIT checklist item numbers. The order of the items has been modified to group similar items (see http://www.equator-network.org/reporting-guidelines/spirit-2013-statement-defining-standard-protocol-items-for-clinical-trials/).

**Table:** 

Title {1}	A pragmatic, open-label, multi-center, randomized controlled clinical trial on the rotational use of interfaces *vs* standard of care in patients treated with noninvasive positive pressure ventilation for acute hypercapnic respiratory failure: the ROTAtional-USE of interface STUDY (ROTA-USE STUDY)
Trial registration {2a and 2b}.	ClinicalTrials.gov identifier: NCT05513508
Protocol version {3}	I version, 1^st^ July 2022 approved by the Ethical Committee of the coordinating center; minor protocol amendment presented on 26^th^ January 2023,was approved by the Ethical Committee on the 21^st^ February 2023.
Funding {4}	This research received no specific grant from any funding agency in the public, commercial or not-for-profit sectors.
Author details {5a}	^1^Dipartimento di Medicina Traslazionale, Università del Piemonte Orientale, via Solaroli, 17, 28100 Novara, Italy ^2^Azienda Ospedaliero-Universitaria Maggiore della Carità, Anesthesia and Intensive Care, Novara, Italy ^3^Department of Surgical, Oncological and Oral Science (Di.Chir.On.S.), University of Palermo, Palermo, Italy ^4^G. Giglio Foundation, Cefalù, Italy ^5^Dipartimento di Medicina e Chirurgia, Università Insubria Varese-Como, Varese, Italy ^6^Department of Anesthesia Analgesia Intensive Care and Emergency. University Hospital Policlinico Paolo Giaccone, Palermo, Italy ^7^Respiratory Medicine Unit, Policlinico “G. Rodolico-San Marco” University Hospital, Catania, Italy ^8^Department of Clinical and Experimental Medicine, University of Catania, Catania, Italy ^9^Respiratory Medicine Unit, Azienda Ospedaliera S. Croce e Carle, Cuneo, Italy ^10^Pulmonology and Respiratory Intensive Care Unit, S. Donato Hospital, Arezzo, Italy ^11^Anesthesia and Intensive Care Unit, Department of Medical and Surgical Sciences, "Magna Graecia" University, Catanzaro, Italy ^12^Dipartimento di Medicina Traslazionele, Università del Piemonte Orientale, Novara, Italy ^13^Dipartimento Scienze mediche, chirurgiche e neuroscienze, Università degli studi di Siena, Siena, Italy ^14^IRCCS Azienda Ospedaliero-Universitaria di Bologna, Respiratory and Critical Care Unit, Bologna, Italy ^15^Dipartimento di Medicina Specialistica, Diagnostica e Sperimentale, Università di Bologna, Bologna, Italy ^16^University Department of Innovative Technologies in Medicine and Dentistry, Università “G. D’Annunzio” di Chieti-Pescara, Italy ^17^Clinical Department of Anesthesiology and Critical Care Medicine, SS. Annunziata Hospital, Chieti, Italy ^18^Dipartimento di Medicina—DIMED—Università di Padova, UOC Istituto di Anestesia e Rianimazione, Azienda Ospedale- Università di Padova, Italy.
Name and contact information for the trial sponsor {5b}	N/A This research received no sponsor
Role of sponsor {5c}	N/A This research received no sponsor

## Introduction

### Background and rationale {6a}

In recent years, noninvasive ventilation (NIV) has increasingly become a common approach for the treatment of hypercapnic [1, 2] and hypoxemic [3] patients both inside [3] and outside [4–6] the intensive care unit (ICU). NIV refers to the delivery of a positive pressure to the patient through interfaces like masks or helmets without the need for invasive endotracheal intubation [7]. These interfaces can efficiently and non-invasively support patients with acute respiratory failure (ARF), preserving airway defense mechanisms, speech, and swallowing, averting the need for prolonged sedation, lowering the occurrence of ventilator-associated pneumonia [8], and ultimately reducing ICU admission [9] and mortality [2]. Nonetheless, NIV failure is observed in 8% of hypercapnic [10] and 50% of hypoxemic patients [11], leading to delayed initiation of invasive ventilation treatment and poor outcomes [5, 10, 12, 13].

As patient comfort is a major factor for NIV success [14], the selection of the most suitable interface type is of paramount importance [15]. Another key variable that should be taken into account to achieve patient comfort and avoid the generation of skin breakdown until pressure ulcers, a frequently observed NIV side effect [7], is the time of exposure to a given interface. Indeed, when the pressure applied between the interface and the skin exceeds the capillary pressure, skin breakdown may occur, resulting in NIV discontinuation due to pain and intolerance [16]. Published incidence rates of facial pressure ulcers associated with NIV masks range from 2 to 31% [17–22] depending on the type of mask used, length of treatment, ventilator setting, humidification, nutritional status, and preventative measures adopted.

The development of pressure ulcers is associated with adverse outcomes that may contribute to patient pain and suffering, impaired quality of life [23], and increased hospital costs [24, 25].

Prevention is usually considered the most efficient solution to address pressure ulcers caused by NIV. Recommended preventative measures for skin damage due to pressure, friction, or shear include the following: (1) correct choice of interface and ventilator (2) reducing the time of uninterrupted NIV administration, (3) application of wound care dressing, and (4) absence of humidification. In particular, the interface should be chosen according to size and attachment. On the one hand, it should not be too loose to cause unintentional leakage, which would worsen patient-ventilator interaction. On the other hand, it should not be too tight to increase the risk of skin breakdown.

As for the exposure time to the interface, a previous study assessing the incidence, location, and stage of pressure ulcers in ICU patients requiring NIV for > 24 h showed that the average exposure time for pressure ulcer development ranged from 1.25 to 74 h ( *x̅* = 28.4 h) for oronasal masks and from 24.75 to 98 h (*x̅* = 61.37 h) for total face masks [17, 18]. The ensuing recommendation was that patients receiving NIV for more than 24 h should be switched to different interfaces to improve their comfort and reduce the occurrence of pressure sores [26–28]. In pre-term infants, the rotational use of interfaces has been tested in randomized controlled trials (RCTs) with discordant results [29, 30]. Among adults, the rotational use of interfaces has been proven feasible both in hypoxemic [28] and hypercapnic [27] patients. Routine interface rotation can also be performed during helmet NIV in a percentage that varies between 3 [31] and 30% [28] in a cohort of patients needing continuous NIV for 1 and 4 days, respectively. Of note, 3% of the patients requiring intermittent helmet continuous positive airway pressure (CPAP) due to COVID-19 needed interface rotation during the treatment [5].

Even though interface rotation is generally accepted as a mean to improve NIV tolerance while reducing the risk of pressure sores [32–34], it is not clear when (i.e., before or after the occurrence of pressure sores) and how often the interface should be rotated. Furthermore, the clinical impact of interface rotation on the incidence or severity of pressure sores and comfort as well as its cost-effectiveness are still unknown.

### Objectives {7}

#### Primary objective

To determine if a protocolized rotational use of interfaces during NIV compared to the standard of care is effective in reducing the development of new pressure sores in patients with hypercapnic ARF treated continuously (> 24 h) with NIV administered (i) to avoid intubation (ii) as an alternative to invasive ventilation, (iii) to avoid reintubation after early extubation, or (iv) to prevent reintubation.

### Trial design {8}

**Fig. 1:**
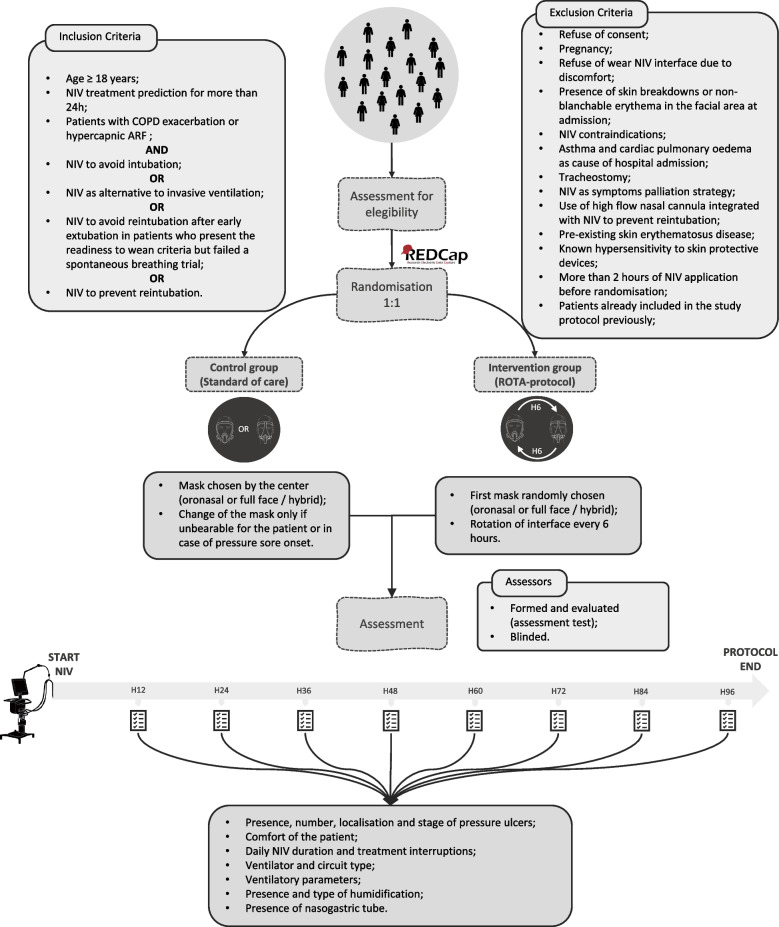
Research design of the study protocol. NIV, noninvasive ventilation; COPD, chronic obstructive pulmonary disease

The ROTA-USE STUDY is designed as a pragmatic, parallel arm, open-label, multicenter, spontaneous, no-profit, randomized, controlled, trial requiring non-significant risk medical devices. Figure 1 summarizes the research design, the main inclusion and exclusion criteria, and the variables collected.

## Methods: participants, interventions, and outcomes

### Study setting {9}

Data will be collected from ICUs, intermediate respiratory care units, respiratory medicine, or internal medicine services in 23 centers in Italy.

### Eligibility criteria {10}

#### Inclusion criteria

To be eligible for randomization, patients must meet all the following criteria:

1. Age ≥ 18 years old;
2. Patients with chronic obstructive pulmonary disease (COPD) exacerbation or with hypercapnic ARF of a different etiology receiving NIV (i) to avoid intubation (pH < 7.35 with an arterial partial pressure of carbon dioxide (PaCO_2_) > 45 mmHg and an arterial partial pressure of oxygen (PaO_2_) < 65 mmHg at room air plus respiratory rate > 25 breaths/min with clinical signs of respiratory muscle distress) or (ii) as an alternative to invasive ventilation (pH ≤ 7.20 with PaCO_2_ > 45 mmHg and PaO_2_ < 65 mmHg at room air plus respiratory rate > 25 breaths/min with clinical signs of respiratory muscle distress) (iii) to avoid reintubation after early extubation in patients who present the readiness to wean criteria but failed a spontaneous breathing trial or (iv) to prevent reintubation;
3. Patients must be admitted to the ICU, intermediate respiratory care unit, respiratory medicine service, or internal medicine service according to the hospital organization; and
4. Treatment forecast must be greater or equal to 24 h.

#### Exclusion criteria

1. Patients, on admission, with skin breakdown or non-blanchable erythema in one of the following areas: nasal bridge, nasolabial fold, cheek, or scalp;
2. Patients who refuse to consent to the study protocol;
3. Patient known to be pregnant;
4. Patients with the following contraindications to NIV: lack of spontaneous breathing; gasping; anatomical or functional airway obstruction; gastrointestinal bleeding or ileus; coma; massive agitation; massive retention of secretions despite bronchoscopy and aggressive physiotherapy; hemodynamic instability (e.g., cardiogenic shock, myocardial infarction); status post-upper gastrointestinal surgery;
5. Patients entering the hospital with asthma or cardiogenic pulmonary edema;
6. Patients with tracheostomy;
7. Patients receiving NIV only for palliation of symptoms (e.g., dyspnea relief). These patients belong to category 3 as defined by the Society of Critical Care Medicine (SCCM) Task Force assessing the palliative use of noninvasive positive pressure ventilation [35];
8. Use of high-flow nasal cannula integrated with NIV to prevent reintubation;
9. Pre-existing skin erythematosus disorders;
10. Known hypersensitivity to skin protective devices (i.e., polyurethane films, colloids, foams);
11. More than 2 h of NIV application before randomization;
12. Patients already included in the study protocol at an earlier stage of their hospital stay; and
13. Refuse to wear NIV interface due to comfort.

### Who will take informed consent? {26a}

Local principal investigators, sub-investigators, trained research nurses, or residents will introduce the trial to the patients or to their legal representatives in case the patients are unable to understand due to communicative or cognitive limitations. The same operators will obtain written consent before randomization from all eligible patients or their legal representatives. Informed consent will be collected according to current national regulations. If patients with legal representatives regain their ability to consent, a second written consent will be requested. In circumstances where patients are unable to consent due to the nature of the underlying disease process (e.g., hypercapnic ARF) or treatments (e.g., sedative medications, mechanical ventilation), in the absence of a legal representative, patients will be randomized, and written consent will be obtained once patients regain their ability to consent. If patients understand and consent to the study, but they are not able to sign a written consent, they will be allowed to verbally agree in the presence of a witness. This verbal consent will then be documented in the medical records. Once the clinical situation improves, the patient will be asked to sign the consent form before being discharged. If a patient dies before a written informed consent can be obtained, his/her data may also potentially be used.

At any time during the clinical study, patients can freely withdraw their informed consent. In this case, no further data will be collected, and the data already collected will be deleted. Participants who abandon the study will be replaced with the aim of reaching the number of subjects envisaged by the sample size, with the possible need to extend the inclusion period.

### Additional consent provisions for collection and use of participant data and biological specimens {26b}

No ancillary studies have been planned so far, and no biological specimens will be collected.

## Interventions

### Explanation for the choice of comparators {6b}

Two groups will be compared. In the control group (i.e., standard-of-care group), interface rotation during NIV will be allowed only in case patients develop pressure ulcer or become intolerant to the interface. In the treatment group, patients will rotate two interfaces, chosen among those used in that center, every 6 h.

### Intervention description {11a}

In the control group, mask will be chosen among the three types of masks available: oronasal, total face, or hybrid mask, according to the standard of care of the participating centers. Patients wearing oronasal masks will receive protective dressing on their nasal bridges before starting NIV. Interface will be changed in case of discomfort, referred by the patient as unbearable, or in the presence of a pressure sore.

In the intervention group, the mask initially applied to the patient will be randomly chosen between the two most used masks in that center among those available (i.e., oronasal, total face, or hybrid mask). Thereafter, NIV mask will be changed every 6 h, alternating the two different interfaces. Patients wearing oronasal masks will receive a protective dressing on the nasal bridge before starting NIV. In case of discomfort, referred by the patient as unbearable related to one of the two interfaces, NIV will be continued with the other mask.

In both arms, the treatment will be continued until patients require NIV according to the assigned protocol.

Prior to patient recruitment, the candidate assessors (doctors and nurses) will be trained to recognize and stage pressure sores through a specific standardized education module. At the end of this course, the trainees will be asked to take a final test where they have to correctly stage 10 pressure sores. Only those trainees able to identify and stage all 10 sores will be qualified as assessors. To minimize detection bias, assessors will not participate in patient care and will be blinded to treatment allocation.

The skin underneath the mask and occipital region will be inspected every 12 h for possible damage by the assessors. If a pressure sore is detected, the physician in charge of the patient will be promptly informed, and a note will be written on the nursing record. Pressure sores will be staged according to the classification of the National Pressure Ulcer Advisory Panel, European Pressure Ulcer Advisory Panel, and Pan Pacific Pressure Injury Alliance [36] (Table 1).

**Table 1:** Pressure sores classification according to the classification of the National Pressure Ulcer Advisory Panel, European Pressure Ulcer Advisory Panel, and Pan Pacific Pressure Injury Alliance

Stage I	A reddened, painful area on the skin that does not turn white when pressed. This is a sign that a pressure ulcer may be forming. The skin may be warm, cool, firm, or soft.
Stage II	The skin blisters or forms an open sore. The area around the sore may be red and irritated.
Stage III	The skin now develops an open, sunken hole called a crater. The tissue below the skin is damaged. It is possible to see body fat in the crater.
Stage IV	The pressure ulcer has become so deep that there is damage to the muscle and bone, and sometimes to tendons and joints.
Unstageable	Is not possible to see the bottom of the sore, and it is impossible to establish how deep it is
Suspected deep tissue injury	The surface of the skin looks like a Stage 1 or 2 sore, but underneath the surface, it appears as a Stage III or IV.

Discomfort due to the mask will be evaluated every 12 h using a 5-level Likert-like scale [17, 37] to answer the questions “How is your level of discomfort related to the mask?”. The extremes of the scale are “1—no discomfort” and “5—unbearable discomfort,” with three intermediate levels: “light discomfort,” “moderate discomfort,” and “intense discomfort”).

In both groups, in case of the presence of a new pressure ulcer, the strategy that can be applied comprehend (1) application of a wound care dressing or a different kind of wound care dressing; (2) reduction of the time spent on NIV; and (3) change interface.

### Criteria for discontinuing or modifying allocated interventions {11b}

Patients will discontinue NIV treatments in case of improvement, worsening leading to intubation, or in case of death.

### Strategies to improve adherence to interventions {11c}

Adherence to interventions will be improved by means of the following strategies:

1. Prior to study protocol initiation, an investigator meeting will be held in each center to detail the study protocol and encourage the staff to adhere to it;
2. The enrolling centers will be selected according to a similar protocol on NIV interface rotation (i.e., in case of the occurrence of a new pressure ulcer or patient discomfort) that represents a common standard of care.

To monitor the adherence to the study protocol, the effective time of ventilation derived from the ventilator counter, the exact time of interface change, and the adherence to the skincare bundle protocol will be recorded as well.

### Relevant concomitant care permitted or prohibited during the trial {11d}

In both arms, diagnostic tests, administration of systemic therapy (e.g., antibiotics, steroids, and inhaled bronchodilators), and hemodynamic management will be performed according to the best clinical practice at enrolling centers. The right size of the mask will be selected according to the indications provided by the manufacturer. The mask straps will be adjusted in compliance with the “two-finger rule” so as to not tighten too hard the headgear [37].

In accordance with international guidelines [1], patients needing NIV to avoid or as an alternative to invasive ventilation will receive NIV for as long as possible during the first 24 h and thereafter until the acute cause is resolved, typically after about 2 to 3 days. NIV will be started when pH < 7.35 and PaCO_2_ > 45 mmHg persist or develop despite optimal medical therapy, targeting an oxygen saturation of 88–92%. Blood gas exchange will be performed before starting NIV treatment, 2 h after NIV institution, and thereafter, when clinically indicated. NIV will be initially set to pressure support mode with a ventilator designed specifically to deliver NIV or with an ICU ventilator equipped with an NIV mode. Positive end-expiratory pressure (PEEP) will be set at 3–5 cmH_2_O, while inspiratory pressure support (PS) will be titrated to achieve adequate augmentation of chest/abdomen movement with an expiratory tidal volume of 6-8 ml/kg and a reduction of respiratory rate (< 30 breaths/min). A good patient-ventilator interaction will be also assessed by checking pressure-flow curve monitoring and the patient’s respiratory pattern. The presence of active humidification will be left to the choice of the patient’s attending physician, although heated humidification should be considered if the patient complains of mucosal dryness or if respiratory secretions are thick and tenacious. The weaning strategy will be documented in the medical and nursing records. The following suggested protocol will be given as a general indication for weaning: continue NIV for 16 h on day 2; 12 h on day 3, including 6–8 h overnight use; NIV may be discontinued on day 4 unless continuation is clinically indicated [26]. The suggested monitoring during NIV will be continuous peripheral oxygen saturation, intermittent measurement of PaCO_2_ and pH, and ECG monitoring in patients with a pulse rate > 120 bpm or with dysrhythmia or possible cardiomyopathy. As the international guidelines recommend keeping the skin in contact with the medical equipment dry and clean, a skincare bundle protocol will be adopted by all centers. If a patient needs prolonged continuous NIV, brakes will be programmed and annotated in the case report form (CRF). In case of instability, the interface will be removed for about 10 min solely to ensure oxygenation [38]. In both groups, when signs of skin breakdown become apparent, strategies involving the application of different wound care dressings, regular breaks whenever possible, and/or rotation between the two interfaces will be implemented. In case of mask-related rash, even in the absence of allergy, topical steroids may be indicated and/or antibiotics if the wound becomes infected. If NIV causes severe gastric distension, a nasogastric tube may be placed.

For patients intubated due to hypercapnic ARF secondary to COPD or other causes related to hypercapnic ARF, NIV might be applied during early extubation and weaning. According to the international guidelines, NIV is recommended to aid weaning from invasive mechanical ventilation, when local expertise in its use exists [1, 14]. Patients receiving invasive mechanical ventilation will be treated according to the clinical guidelines [1]. After reaching maximum medical treatment, once the ventilation is set to controlled mode and pH and PaCO_2_ are normalized in the presence of neurological, hemodynamic, and respiratory stability, patients will be extubated and subjected to NIV. The ventilator will be initially set at the same PEEP and inspiratory PS level applied during invasive mechanical ventilation, setting it at the fastest pressure rise time. Successively, NIV setting will be titrated to maintain arterial blood gas in a normal range, a tidal volume between 6 and 8 ml/kg of predicted body weight, and a respiratory rate below 25 breaths/min. In patients tolerating spontaneous breathing. NIV will be gradually tapered off until they can permanently sustain spontaneous breathing.

### Provisions for post-trial care {30}

NIV is extensively used in clinical practice. All patients included in the study deserve NIV treatment according to their clinical symptoms and pathology. Major complications due to NIV treatment are not expected. If any complications occur, they will be treated according to the best clinical practice.

### Outcomes {12}

The primary effectiveness outcome is the proportion of patients developing at least one pressure sore of any stage at 36 h from randomization. The reason why this measure of effectiveness was chosen is that it is more clinically relevant than the overall number of pressure sores. Furthermore, 36 h seems to be an appropriate time point given that the time it takes for pressure sores to develop is extremely variable, but it might as well be very short.

Secondary outcomes will be the onset, number, and stage of pressure sores and comfort during NIV measured at different time points (12, 24, 36, 48, 60, 72, 84, 96 h). At the same time points, the location of pressure sores per patient will also be recorded, i.e., forehead, nose bridge, left cheek, right cheek, chin, left periauricular, right periauricular, and occipital. The study will also monitor the number of patients that interrupts NIV due to discomfort, the presence/absence of eye irritation in relation to both oronasal and full face/hybrid mask, poor adherence to the protocol (e.g., duration of NIV treatment/day, interfaces changed and hygiene protocol application both during each 12-h protocol period) and the presence/absence and types of vasoactive medications, i.e., epinephrine, norepinephrine, vasopressin, dobutamine, dopamine, other. Further outcomes include the costs for intervention implementation (e.g., mask-related costs) and those related to the care of pressure sores (antibiotic treatment, specialist medical visits).

### Participant timeline {13}

**Fig. 2:**
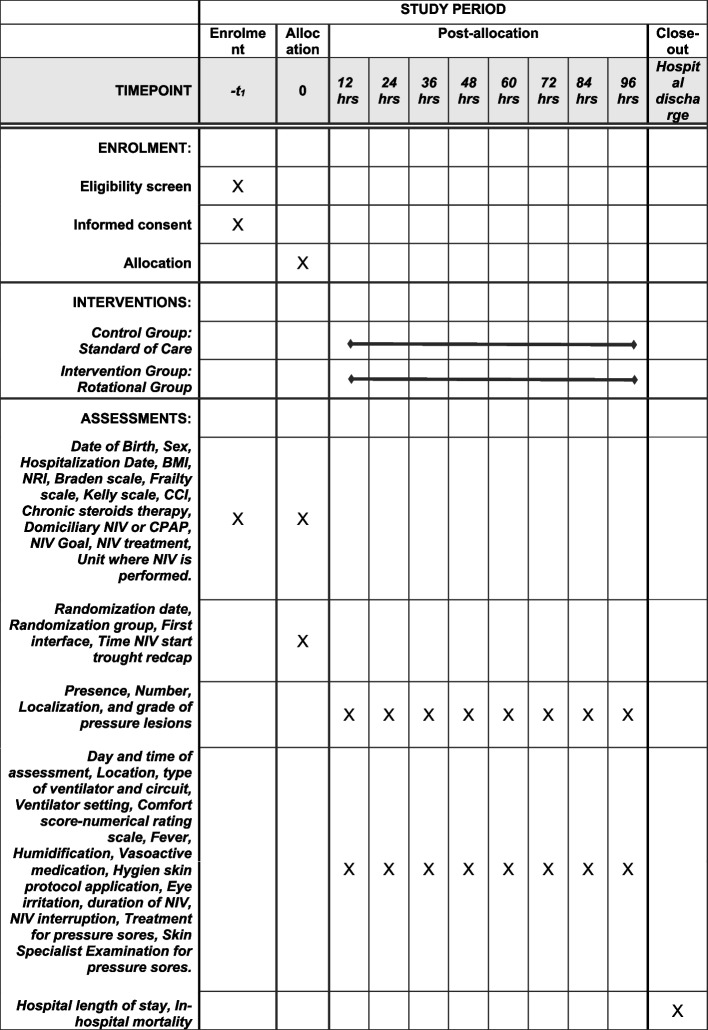
Schedule of enrolment, interventions, and assessments. BMI, body mass index, NRI, Nutritional Risk Index; CCI, Charlson Comorbidity Index; NIV, noninvasive ventilation; CPAP, continuous positive airway pressure

Patients will be screened for eligibility criteria. Consent will be obtained from the patients, if possible, or their legal representative. Thereafter, patients will be randomized into the treatment or control group. After randomization, the skin underneath the mask and the auricle and occipital regions will be inspected to check for any possible pressure sores every 12 h for the following 4 days. Patient status at hospital discharge will also be evaluated (Fig. 2).

### Sample size {14}

Given a type I error of 0.05, a power of 0.8, an expected proportion of patients who develop pressure sores in the control group of 23% [39], and an expected dropout rate of 5%, 239 subjects per group are estimated to identify a 10% absolute difference in the proportion of patients developing pressure sores between intervention and control groups measured at 36 h from randomization.

### Recruitment {15}

A survey was administered during the planning phase of the protocol to identify possible recruiting centers and estimate the number of patients that each center could possibly enroll in the study period. Thereafter, the list of the participating centers was redacted and proposed. In case a problem of low recruitment is encountered, any center with a consistently low recruitment rate will be contacted to address site-specific issues. If necessary, new investigators and sites will be added to overcome low recruitment rates.

## Assignment of interventions: allocation

### Sequence generation {16a}

Computer-based random sequences will be generated by the statistician in charge of the analyses. A total of 240 random extractions from a Bernoulli distribution with a probability equal to 0.5 will be performed to assign patients 1:1 to the interface rotation (treatment) or standard of care (control) group. Moreover, following the same procedure, patients in the interface rotation group will be assigned to start their treatment wearing an oronasal mask or full face/hybrid masks. The randomization list will be uploaded into the research electronic data capture (REDCap) system that will provide the assigned arm of the patients. Investigators will have no access to the randomization list.

### Concealment mechanism {16b}

To ensure allocation concealment, the randomization list will be uploaded directly into REDCap. Allocation will take place after the patient is recruited to the trial.

### Implementation {16c}

The statistician (LS) will prepare the randomization list that will be uploaded into REDCap. Once the staff members enroll a new patient, he/she will access REDCap that will provide the randomization arm and mask sequence after filling out the inclusion criteria form.

## Assignment of interventions: blinding

### Who will be blinded {17a}

Due to the nature of the intervention tested, trial participants and care providers will not be blinded after being assigned to an intervention. However, assessors and data analysts will not participate in the care of the patient and will be blinded to patient group assignment.

### Procedure for unblinding if needed {17b}

Unblinding procedure is not required.

## Data collection and management

### Plans for assessment and collection of outcomes {18a}

A REDCap database will be set up to collect the information needed for the study. The database will consist of ten sheets. The first one will contain the baseline demographic and clinical characteristics of the patients. Eight sheets will store information on primary and secondary outcomes at each time point. The last one will enlist information on patient discharge and the overall treatment. More specifically, the following variables will be recorded:

- *Baseline characteristics*: age, sex, hospitalization date, nutritional status, body mass index, Braden scale, frailty index, sensorium (Kelly score), Charlson index, chronic steroids therapy, domiciliary NIV or CPAP, NIV treatment goal.
- *Outcome assessment:* presence and number of pressure sores, location, comfort score (numerical rating scale from 1 to 5), stage for each lesion, fever, humidification, vasoactive medication, NIV interruption, daily NIV duration, hygiene protocol application, eye irritation, interface change, time interface change, NIV ventilator, circuit type, expiratory positive airway pressure, inspiratory positive airway pressure, leaks, medical treatment for the pressure lesions and eventual skin specialist consult.
- *Discharge:* discharge date, length of hospital stay, discharge status.

### Plans to promote participant retention and complete follow-up {18b}

Once a patient is enrolled or randomized, the study investigators will make every reasonable effort to follow the patient for the entire study period. This trial does not entail a long follow-up period, thus the rate of loss-to-follow-up is anticipated to be negligible.

### Data management {19}

Data entry will be carried out by the investigators of each study center after the participation in training session before the beginning of the study to standardize procedures, including data collection.

Investigators may contact the coordinating center to solve issues that may arise.

Only authorized staff will be able to access the REDCap system. A two-step login procedure is needed to access the system. The first step requires the login with username and password. After the completion of the first step, an e-mail is sent to the user by the system with a verification code to be entered in the second step login, which expires after two minutes.

Investigators will only be granted access to the records of their own patients but not to those stored in the entire database.

The data will be automatically stored by REDCap, with backups planned every 4 h.

During the creation of data collection sheets, when possible, the data format and range of plausible values will be set. If the input value is outside the defined range, a warning message will automatically appear to the researcher.

Data will first be collected with a paper support and, after having checked for completeness and consistency, will be entered in REDCap.

After the end of data collection:

- Data provided by the centers will be checked for missing data, plausible, possible, or non-permitted value ranges. When necessary, centers will be asked to correct them;
- Statistical techniques to identify inconsistencies will be applied periodically. The centers will be notified about eventual inconsistencies to correct them;
- The coordinating center will review detailed reports on screening, inclusion, follow-up, and data consistency and completeness. The coordinating center will take immediate action to solve any problems that may arise.
- Data will be entered in the final electronic database only after the CRFs have been cleared by the coordinating center.

### Confidentiality {27}

The electronic CRF, which will not include any names, initials, dates of birth, or local hospital patient numbers, is identified by a unique subject identifier. Data protection in the database will be guaranteed through encoding and the use of a secured database with restricted access by individual login and gradated user rights. Lastly, only encrypted data will be centrally stored.

### Plans for collection, laboratory evaluation, and storage of biological specimens for genetic or molecular analysis in this trial/future use {33}

No biological specimens will be collected.

## Statistical methods

### Statistical methods for primary and secondary outcomes {20a}

The analysis will be performed according to the intention-to-treat (ITT) principle. Descriptive statistics will be calculated to summarize patient characteristics collected at study entry according to the randomization group. Categorical variables will be reported as absolute frequencies and percentages. Continuous variables will be represented as mean and standard deviation or median first and third quartiles if not normally distributed according to the Shapiro–Wilk test.

The raw risk of pressure sore will be calculated at each time point as the ratio between the number of subjects who developed a pressure sore at a given time point divided by the number of subjects undergoing NIV at that time point. Patients having a planned break at the time point considered, but not weaned off NIV, will be considered as patients undergoing NIV.

Mixed effect logistic regression models will be used to estimate the risk of pressure sores and the difference between interfaces, including a random intercept to account for clustering of the subjects within the center and repeated measures within patients. The response variable will be the presence/absence of pressure sores, whereas the independent variables will be the treatment, time (12, 24, 36, 48, 60, 72, 84, 96 h), and their interaction. The marginal predicted probabilities estimated by the model will provide the incidence of pressure sores at different times as well as overall. The inclusion of time as a covariate will allow to test the presence of a time trend in the incidence of pressure sores and to investigate the variation of time trends according to treatment group.

If the descriptive statistics will detect unbalanced characteristics between randomization groups (e.g., indication for NIV), the corresponding variables will be added as covariates in the logistic regression model.

Regarding the number of pressure sores, different mixed effect Poisson regression models will be applied: one for each time point to estimate the average number of pressure sores at each time point and a model in which only the highest number of pressure sores will be considered for each patient. Time will be included as offset to evaluate the average number of pressure sores per day.

The grade and location of pressure sores will be summarized using descriptive statistics at each time point.

The analyses will be also performed according to the modified intention-to-treat approach (mITT) excluding subjects with a NIV duration shorter than 12 h to ensure patients in the rotational group performed at least one rotation of the interface.

Regarding the economic evaluation, the incremental cost-effectiveness ratio (ICER) will be calculated considering two outcomes: (i) the proportion of pressure sores and (ii) the patient comfort. ICER, which is the ratio between the difference of each outcome in the two groups (i.e., difference in the proportion of pressure sores or comfort score) and the difference in the average cost for each treatment will allow to evaluate the incremental costs needed to reduce of 1 point the outcomes thanks to interface rotation. The confidence intervals of the ICER will be calculated using the non-parametric bootstrap method. Specifically, the 2.5^th^ and 9.75^th^ percentiles of the bootstrap distribution of ICER will be considered as the lower and higher limits of the confidence interval. The cost-effectiveness acceptability curve will also be drawn to evaluate the probability of the intervention to be cost-effective, considering different thresholds.

### Interim analyses {21b}

A single interim analysis will be performed when two-thirds of the predefined sample are randomized and completed the study. Early stopping criteria will be a predetermined 2-sided *p*-value < 0.01 for the rejection of the null hypothesis that the strategies are equivalent in terms of pressure sore incidence rate.

### Methods for additional analyses (e.g., subgroup analyses) {20b}

When possible (i.e., enough patients are available), the analyses will be performed stratified by center.

### Methods in analysis to handle protocol non-adherence and any statistical methods to handle missing data {20c}

Protocol non-adherence will be handled by performing the analyses according to the ITT principle, specifically, patients will be analyzed considering their allocation group regardless of the intervention they will follow. Moreover, deviation from the protocol will be registered as well as the reason for deviation. The missing rate is expected to be very low, therefore no specific analyses to handle missing data are planned. However, if needed (i.e., missing rate between 20 and 40%) multiple imputation through expectation–maximization algorithm will be applied and the results obtained from analysis of the new data will be considered as sensitivity analysis.

### Plans to give access to the full protocol, participant-level data, and statistical code {31c}

Public access to the full protocol and statistical methods will be obtained by publishing the study protocol in an open-access journal. Study protocol has been submitted to ClinicalTrials.gov (identifier: NCT05513508).

## Oversight and monitoring

### Composition of the coordinating center and trial steering committee {5d}

The coordinator center is based at the Università del Piemonte Orientale and includes Rosanna Vaschetto (principal investigator), Nello De Vita (research physician), Eduardo Rocca (research physician), Alberto Dal Molin (research nurse) e Lorenza Scotti (methodologist). Coordinator center tasks will consist in preparing protocol and revisions; designing CRFs; organizing steering committee meetings; managing and controlling data quality; designing, testing, and maintaining the electronic data capture system; assisting the steering committee; managing the research centers; selecting and training the research centers; helping the centers prepare all the necessary documents to be submitted to research ethics boards (REBs) and assisting centers with submission; monitoring recruitment rates; sending study materials to the research centers; complete statistical analysis; and writing and publication of study protocol and final manuscript.

The trial steering committee is composed by Cesare Gregoretti (lead investigator), Annalisa Carlucci (lead investigator), Andrea Cortegiani (lead investigator), Claudia Crimi (lead investigator), Alessio Mattei (lead investigator), Raffaele Scala (lead investigator), Federico Longhini (lead investigator), Gianmaria Cammarota (lead investigator), Giovanni Misseri (lead investigator), and Paolo Navalesi (senior lead investigator). The trial steering committee responsibilities will be agreement on the final protocol, recruitment of patients and liaising with the principal investigator, reviewing the progress of the study, and if necessary agreeing on changes to the protocol and/or investigators brochure to facilitate the smooth running of the study, agreement on the final manuscripts.

### Composition of the data monitoring committee, its role and reporting structure {21a}

The data monitoring group is composed by Stefano Nava (lead investigator), Sabino Scolletta (lead investigator), and Salvatore Maurizio Maggiore (lead investigator). The role of the data monitoring committee is to independently review the study protocol, recruitment of patients and liaising with the principal investigator to be involved in the safety monitoring interim analysis, and agreement on the final manuscripts. The Data Quality Assurance group is composed by Eduardo Rocca, Federico Longhini, Gianmaria Cammarota, Giovanni Misseri, and Alberto Dal Molin and will be responsible for monitoring data for inconsistency and missing data.

### Adverse event reporting and harms {22}

Interfaces for noninvasive ventilation should be considered as a non-significant risk device according to the definition given from the Food and Drug Administration. Besides, the present randomized controlled trial does not intend to investigate a new interface but the protocolized rotational use of commercially available interfaces. As patients with contraindications to NIV/with known hypersensitivity to skin protective devices will be excluded from the study, the potential for serious adverse events related to a protocolized rotation use of interfaces appears remote. Possible minor adverse events consequent to the use of noninvasive ventilation interfaces consist of pressure sores, dryness of the mouth, eye redness, and discomfort. All these information will be recorded and reported as primary and secondary outcomes of the study.

### Frequency and plans for auditing trial conduct {23}

At the start of the trial, the coordinating center will conduct a tutorial on the web-based data entry system. Overall quality and completeness of the data will be checked every six months.

### Plans for communicating important protocol amendments to relevant parties (e.g., trial participants, ethical committees) {25}

Any modifications to the protocol that may impact the conduct of the trial will require a formal amendment to the protocol. All amendments to the protocol must be approved by the internal review board of each participating center. Administrative changes to the protocol, minor corrections, and/or clarifications that have no effect on the way the trial is to be conducted will be agreed upon among the participating centers.

### Dissemination plans {31a}

The ROTA-USE STUDY group will publish the study findings, whatever they are. The main manuscript will be submitted by the writing committee on behalf of the research group. The principal investigators of the three top enrolling centers will be included and engaged in the writing process. Collaborative authorship (i.e., group author under the name of ROTA-STUDY group) will be offered to the principal investigators and research nurses of all the centers including at least 20 patients.

## Discussion

All the issues have been covered in the above sections.

## Trial status

The ROTA-USE protocol, version I (01/07/22), has been approved by the research ethics committee of the coordinator center on the 16/09/22, and recruitment has started in December 2022 at the coordinating center. The remaining centers are undergoing ethics committee evaluation. Recruitment is expected to be completed within September 2024.

## Data Availability

Access to the database will be restricted to the coordinating team, and third parties will only have access to it upon receiving the authorization from the principal investigator, who will analyze each research proposal and statistical analysis plan.

## Abbreviations

ARF: Acute respiratory failure
COPD: Chronic obstructive pulmonary disease
CPAP: Continuous positive airway pressure
CRF: Case report form
PEEP: Positive end-expiratory pressure
ICER: Incremental cost-effectiveness ratio
ICU: Intensive care unit
ITT: Intention-to-treat
mITT: Modified intention-to-treat
NIV: Noninvasive ventilation
PaCO_2_: Arterial partial pressure of carbon dioxide
PaO_2_: Arterial partial pressure of oxygen
PS: Pressure support
RCT: Randomized controlled trial
REB: Research ethics board
